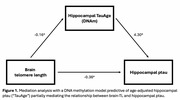# Brain telomere length associates with hippocampal ptau and is mediated by DNA methylation

**DOI:** 10.1002/alz70855_105996

**Published:** 2025-12-24

**Authors:** Nadia Dehghani, Qijun Chen, Anil R. Wadhwani, David C. Goldberg, Jin Jin, Lasya P. Sreepada, Isabella Leone, Angelina Jala, David A. Wolk, Eddie B. Lee, Kurt Farrell, John F. Crary, Wanding Zhou, F. Bradley Johnson, Corey T. McMillan, PART Working Group

**Affiliations:** ^1^ Department of Neurology, University of Pennsylvania Perelman School of Medicine, Philadelphia, PA, USA; ^2^ Department of Pathology & Laboratory Medicine, University of Pennsylvania Perelman School of Medicine, Philadelphia, PA, USA; ^3^ Center for Computational Genomic Medicine, Children's Hospital of Philadelphia, Philadelphia, PA, USA; ^4^ Department of Biostatistics, Epidemiology and Informatics, University of Pennsylvania Perelman School of Medicine, Philadelphia, PA, USA; ^5^ Department of Bioengineering, University of Pennsylvania School of Engineering and Applied Sciences, Philadelphia, PA, USA; ^6^ Department of Neurology, Perelman School of Medicine, University of Pennsylvania, Philadelphia, PA, USA; ^7^ Department of Pathology & Laboratory Medicine, Perelman School of Medicine, University of Pennsylvania, Philadelphia, PA, USA; ^8^ Icahn School of Medicine at Mount Sinai, New York, NY, USA

## Abstract

**Background:**

Telomeres are repetitive DNA sequences at the ends of chromosomes which contribute to maintaining chromosomal stability. Telomere shortening is a hallmark of aging and shorter blood leukocyte telomere length (LTL) has been associated with increased risk for age‐related diseases, however, little is understood about the biology of brain telomeres and how they may be involved in disease. Considering the increased neuropathologic burden of phosphorylated tau (ptau) with age, we investigated how shorter brain telomere length (brain‐TL) may relate to increased ptau burden.

**Methods:**

We studied a cohort of 112 individuals with primary age‐related tauopathy (PART), a neuropathological diagnosis characterized by mild‐to‐moderate tau burden (Braak=I‐IV) primarily in the medial temporal lobe, with the relative absence of amyloid‐beta plaques (CERAD=0). These individuals had both brain‐TL (mean length by telomere qPCR, blinded) and DNA methylation measures from the frontal cortex, along with semi‐quantitative Aperio ptau measures from the hippocampus. A subset (*n* = 81) had SNP genotyping data available. In an independent cohort (*n* = 10, Braak=0‐VI, CERAD=0‐3), we performed quantitative fluorescence *in‐situ* hybridization (FISH) microscopy to measure the average ratio of telomere to centromere DNA content in nuclei from the frontal and visual cortices.

**Results:**

In linear regression models, frontal cortex brain‐TL did not relate to age. When age‐adjusted, shorter brain‐TL related to higher hippocampal ptau (β=‐1.06, CI=‐1.92–‐0.195, *p* = 0.017). A previously established DNA methylation model predictive of hippocampal ptau partially mediated the relationship between brain‐TL and hippocampal ptau (proportion mediated=0.664, CI=0.246–1.33, *p* = 0.012, Figure 1). A polygenic score for LTL did not relate to either age, brain‐TL or hippocampal ptau. With FISH, we observed that individuals with CERAD=0 had shorter telomeres in the frontal cortex compared to individuals with CERAD=3. Within the CERAD=0 group, an individual with Braak=II had shorter telomeres than an individual with Braak=I. These patterns were not observed in the visual cortex.

**Conclusions:**

In a PART cohort, shorter frontal cortex brain‐TL was related to higher hippocampal ptau, and this relationship was partially mediated by a DNA methylation model predictive of hippocampal ptau. A polygenic score for LTL was not predictive of brain‐TL or hippocampal ptau. Together, this further emphasizes the importance of tissue‐specific epigenetic modifiers of age‐related ptau neuropathology.